# A structurally informed autotransporter platform for efficient heterologous protein secretion and display

**DOI:** 10.1186/1475-2859-11-85

**Published:** 2012-06-18

**Authors:** Wouter SP Jong, Zora Soprova, Karin de Punder, Corinne M ten Hagen-Jongman, Samuel Wagner, David Wickström, Jan-Willem de Gier, Peter Andersen, Nicole N van der Wel, Joen Luirink

**Affiliations:** 1 Section Molecular Microbiology, Department of Molecular Cell Biology, Faculty of Earth and Life Sciences, VU University, De Boelelaan 1085, 1081 HV, Amsterdam, The Netherlands; 2 Xbrane Bioscience, SE-111 45, Stockholm, Sweden; 3 The Netherlands Cancer Institute, Antoni van Leeuwenhoek Hospital, 1066 CX, Amsterdam, The Netherlands; 4 Inter-Faculty Institute of Microbiology and Infection Medicine Tübingen (IMIT), 72076, Tübingen, Germany; 5 Center for Biomembrane Research, Department of Biochemistry and Biophysics, Stockholm University, SE-106 91, Stockholm, Sweden; 6 Department of Infectious Disease Immunology, Statens Serum Institut, Copenhagen, Denmark

**Keywords:** Autotransporter, Type V secretion, Hemoglobin protease, Extracellular expression, Surface display, Live vaccine

## Abstract

**Background:**

The self-sufficient autotransporter (AT) pathway, ubiquitous in Gram-negative bacteria, combines a relatively simple protein secretion mechanism with a high transport capacity. ATs consist of a secreted passenger domain and a β-domain that facilitates transfer of the passenger across the cell-envelope. They have a great potential for the extracellular expression of recombinant proteins but their exploitation has suffered from the limited structural knowledge of carrier ATs. Capitalizing on its crystal structure, we have engineered the *Escherichia coli* AT Hemoglobin protease (Hbp) into a platform for the secretion and surface display of heterologous proteins, using the *Mycobacterium tuberculosis* vaccine target ESAT6 as a model protein.

**Results:**

Based on the Hbp crystal structure, five passenger side domains were selected and one by one replaced by ESAT6, whereas a β-helical core structure (β-stem) was left intact. The resulting Hbp-ESAT6 chimeras were efficiently and stably secreted into the culture medium of *E. coli*. On the other hand, Hbp-ESAT6 fusions containing a truncated β-stem appeared unstable after translocation, demonstrating the importance of an intact β-stem. By interrupting the cleavage site between passenger and β-domain, Hbp-ESAT6 display variants were constructed that remain cell associated and facilitate efficient surface exposure of ESAT6 as judged by proteinase K accessibility and whole cell immuno-EM analysis. Upon replacement of the passenger side domain of an alternative AT, EspC, ESAT6 was also efficiently secreted, showing the approach is more generally applicable to ATs. Furthermore, Hbp-ESAT6 was efficiently displayed in an attenuated *Salmonella typhimurium* strain upon chromosomal integration of a single encoding gene copy, demonstrating the potential of the Hbp platform for live vaccine development.

**Conclusions:**

We developed the first structurally informed AT platform for efficient secretion and surface display of heterologous proteins. The platform has potential with regard to the development of recombinant live vaccines and may be useful for other biotechnological applications that require high-level secretion or display of recombinant proteins by bacteria.

## Background

Despite their complex cell envelope, Gram-negative bacteria are widely used for the extracellular expression of proteins to facilitate downstream processing and to improve biological activity, solubility and stability [1]. For specific applications, delivery and attachment of recombinant proteins or peptides at the cell surface is preferred over full secretion [2-4]. Examples include live vaccines, biocatalysis and high throughput screening of peptide libraries for drug discovery.

Several pathways have evolved for transfer of proteins across the multipart cell envelope of Gram-negative bacteria that consists of an inner (IM) and outer membrane (OM), separated by the periplasm that contains a mesh-like peptidoglycan layer. The autotransporter (AT) pathway, a branch of the type V secretion system, is the most common and simple mechanism, which is typically used for the secretion of large virulence factors [5]. ATs comprise three domains: (i) an N-terminal signal peptide that targets the protein to the Sec translocon for translocation across the IM, (ii) a passenger domain, which is the actual secreted moiety, and (iii) a C-terminal β-domain that integrates into the OM and facilitates translocation of the passenger from the periplasm into the extracellular space. Recent evidence indicates that the AT pathway is not entirely self-sufficient and requires the Bam complex for passenger translocation across the OM [6,7]. Potential application of ATs for extracellular expression of recombinant proteins has been recognized from the time the first AT was characterized [8]. However, successful exploitation of the system was hampered by limited structural knowledge of the carrier ATs [2,3].

Previously, we have presented the crystal structure of the secreted passenger of the *Escherichia coli* AT Hemoglobin protease (Hbp) [9]. The structure revealed a long β-helical core domain (β-stem) that appears to function as a stable scaffold for five side domains (d1-d5) (Additional file 1: Figure S1) [9]. The β-stem is well conserved among ATs and the stepwise stacking of the β-strands at the cell surface has been suggested to provide a pulling force or Brownian ratchet for vectorial transport across the OM [10-14]. A conserved region at the C-terminus of the passenger domain, the so-called autochaperone (AC) domain, has recently been implicated in the initiation of this process [13,15].

Here, we developed a structurally informed Hbp-based platform for the secretion and surface display of heterologous proteins using the *Mycobacterium tuberculosis* antigen and vaccine target ESAT6 [16] as a model protein. More specifically, we identified sites in the Hbp passenger domain that are permissive with respect to the insertion of ESAT6. It is shown that substitution of Hbp passenger side domains is a successful strategy to achieve high-level secretion and display of ESAT6 in *E. coli*. Although not essential for the translocation process *per se*, an intact Hbp β-stem appeared important to achieve optimal secretion and stability of Hbp-ESAT6 chimeras. To demonstrate the general applicability of this approach, ESAT6 substituting a side domain of the passenger domain of another AT, EspC, was found to be efficiently secreted. Furthermore, we show stable and efficient display of ESAT6 fused to Hbp in an attenuated *Salmonella typhimurium* strain upon chromosomal integration of the encoding gene, demonstrating the potential in the development of bacterial live vaccines.

## Results and discussion

### Building an Hbp-based platform for the secretion of heterologous proteins

**Figure 1:**
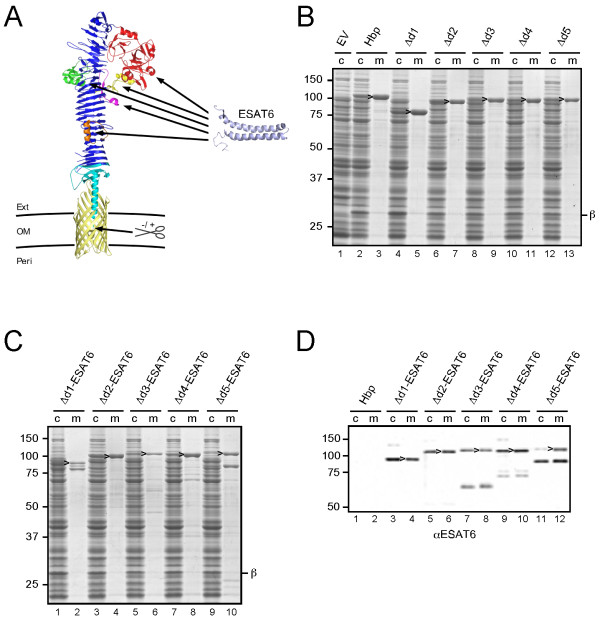
**Secretion of ESAT6 fused to the passenger of Hbp.** (**A**) Schematic representation of secretion and display strategy based on the Hbp passenger and β-domain crystal structures [9,44]. ESAT6 [45] is fused to the Hbp passenger domain by replacing any of the side domains d1 (*red*), d2 (*green*), d3 (*yellow*), d4 (*magenta*) or d5 (*orange*). Scissors indicate a cleavage site between the passenger and β-domain, which was left intact (+) for secretion purposes and disrupted (-) for surface display. The image was created using MacPyMol. (**BC**) Hbp constructs were expressed in *E. coli* MC1061 and the equivalent of 0.03 OD_660_ units cells (c) and corresponding culture medium (m) samples was analyzed by SDS-PAGE and Coomassie staining. (**B**) Expression and secretion of Hbp constructs compared to the empty vector control (EV). (**C**) Secretion of Hbp constructs carrying ESAT6. (**D**) Samples described under *C* were analyzed by immunoblotting using ESAT6 specific antibodies. Cleaved Hbp passenger (>) and ~ 28 kDa β-domain [19] (*β*) are indicated. Molecular mass (kDa) markers are shown at the left side of the panels.

The available crystal structure of the secreted Hbp passenger domain [9] allows the design of translational fusions with minimal perturbation of the native structure. Whereas the conserved β-stem of AT passengers has been implicated in biogenesis and transport [11,12], the Hbp passenger side domain d2 appeared dispensable for secretion of Hbp [9,17]. Hbp in which d2 had been replaced by human Calmodulin was efficiently secreted provided that Ca^2+^ was chelated in the medium to prevent folding of calmodulin into a rigid translocation-incompetent conformation. Furthermore, the incorporation of a disulfide bonded cysteine pair in side domain d4 was tolerated during secretion [17]. Therefore, we focused on side domains as potential sites for the integration of heterologous polypeptides (see Figure 1A).

To verify that d1-d5 are dispensable for secretion, they were replaced by a small flexible spacer of alternating glycine and serine residues (Additional file 2: Figure S2). The resulting Hbp-derivatives were cloned under *lac*-promoter control and expressed in *E. coli* strain MC1061. Cells were grown to early log-phase after which expression of the Hbp was induced by the addition of isopropyl β-D-thiogalactopyranoside (IPTG) and growth was continued for 2 h. Samples were then collected and centrifuged to separate cells and spent medium. To monitor expression and secretion of Hbp, both fractions were analysed by SDS-PAGE and Coomassie staining (Figure 1B). Deletion of d1 did not affect expression, processing and secretion as shown by the appearance of cleaved passenger in cell and medium fractions, and cleaved β-domain in the cell fraction at a level comparable to wild-type Hbp (Figure 1B, lanes 2-5) [18,19]. Removal of d2-d5 was slightly less well tolerated causing a reduction in expression level and, hence, amounts of secreted Hbp of up to ~50% compared to wild-type Hbp (Figure 1B, lanes 6-13). The identity of passenger and β-domain species was confirmed by immunoblotting (data not shown). In conclusion, the passenger side domains d1-d5 are dispensable for the Hbp secretion process.

To investigate the potential of Hbp to secrete sizeable heterologous proteins, side domains d1-d5 were replaced one by one by the 9.9 kDa secretory *M. tuberculosis* antigen ESAT6 [16], which was previously suggested to attain an α-helical hairpin conformation when fused to the Hbp passenger [20] (see Figure 1A). The antigen was inserted into the glycine/serine spacers that replaced the side domains (see above) to ensure optimal conformational flexibility with respect to the β-stem (Additional file 2: Figure S2). Protein staining (Figure 1C) and immunoblotting (Figure 1D) showed efficient secretion of all Hbp-ESAT6 chimeras although truncated passengers were detected possibly due to proteolytic cleavage (Figure 1C lanes 2 and 10; Figure 1D, lanes 7-12). We conclude that replacement of passenger side domains is a successful strategy to achieve high-level secretion of ESAT6.

Previous work [17,21] suggests that proteins expected to form multiple disulfide bonds or to attain a rigid bulky structure in the periplasm are largely incompatible with translocation through the Hbp system and prone to degradation by the periplasmic protease DegP [17]. This problem can be overcome by adapting growth conditions and host background to preclude disulfide bond formation or tight folding [17,21,22]. Alternatively, the fusion partner itself can be modified e.g. by removing cysteine residues (Additional file 3: Figure S3, cf. hEGF and hEGF[0ss]). Interestingly, the immunoglobulin G (IgG) binding ZZ domain of protein A from *Staphylococcus aureus* appeared fully functional in IgG binding when displayed at the *E. coli* cell surface by Hbp (data not shown), demonstrating the compatibility of our system with post-translocational folding of fused proteins.

### An intact β-stem is important for extracellular expression of Hbp-ESAT6

**Figure 2:**
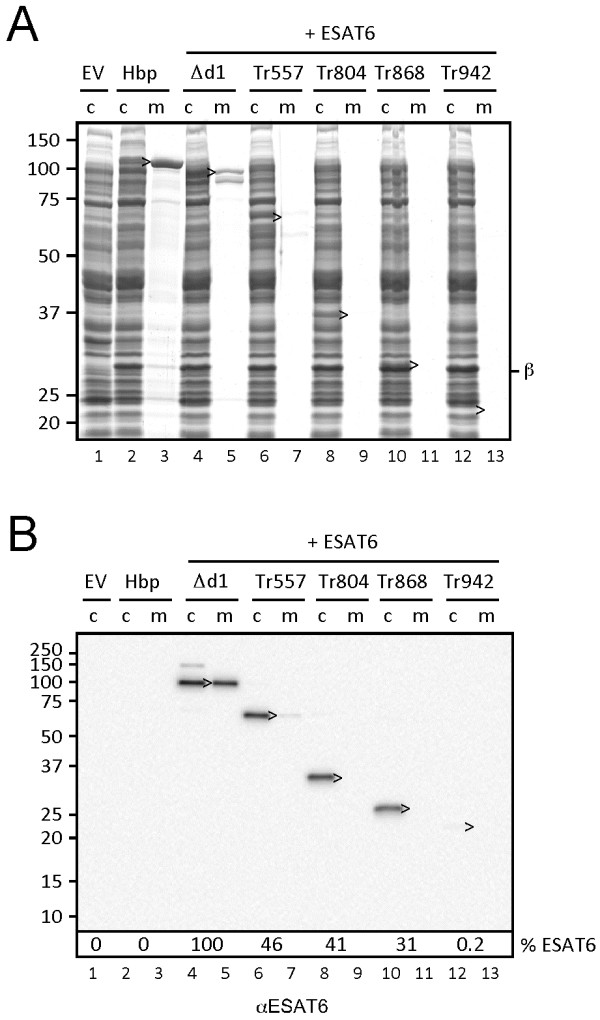
**Secretion of ESAT6 upon fusion to a truncated Hbp passenger.** (**A**) Secretion of Hbp constructs compared to the empty vector control (EV), analyzed by SDS-PAGE and Coomassie staining as described in the legend to Figure 1. (**B**) Analysis of the samples described under *A* by immunoblotting using ESAT6 specific antibodies. The amount of ESAT6 recovered from the respective cultures was quantified and displayed at the bottom of the panel as relative amounts (%) compared to Hbp(Δd1)-ESAT6. Molecular mass (kDa) markers are indicated at the left side of the panels. Cleaved passenger (>) is indicated.

Although for most applications the presence of the core β-helix structure of the Hbp passenger in chimeric proteins offers no conceptual problem, a shorter product may reduce the metabolic burden in critical biotechnological processes. In previous studies, however, fusion of heterologous proteins to strongly truncated AT passengers, or even directly to AT β-domains, often yielded inefficient transport to the extracellular environment [2]. Here, we systematically analyzed the relationship between the length of the Hbp passenger and its functionality as a fusion partner for secretion and display of heterologous proteins. To this end, the secretion of Hbp(Δd1)-ESAT6, in which a complete β-stem is still present, and four derivatives containing progressively truncated passengers (Additional file 2: Figure S2) was analyzed as described above using SDS-PAGE and Coomassie staining (Figure 2A) or immunoblotting (Figure 2B).

N-terminal fusion of ESAT6 to an Hbp passenger that was truncated down to residue 557 (HbpTr557), just downstream of side domain d2, resulted in a ~2-fold decrease in the recovery of cleaved Hbp-ESAT6 passenger material from total culture samples compared to Hbp(Δd1)-ESAT6 (Figure 2A, lanes 4-7; Figure 2B, lanes 4-7). Upon further truncation, the amount of recovered passenger decreased even further down to only ~0.2% relative to Hbp(Δd1)-ESAT6 for the shortest construct Hbp(Tr942)-ESAT6, corresponding to fusion of ESAT6 to the AC domain of the Hbp passenger (Figure 2A, lanes 8-13; Figure 2B, lanes 8-13). For all truncates hardly any passenger material was recovered from the spent medium fraction suggesting that truncation of the passenger also interferes with release from the cell surface (Figure 2A, lanes 7,9,11,13; Figure 2B, lanes 7,9,11,13). Notably, similar amounts of cleaved β-domain accumulated in all cell fractions (Figure 2A) arguing that the Hbp-ESAT6 truncates are expressed, translocated across the OM and cleaved. Most likely, during or after translocation the truncated passenger-ESAT6 fusions are unstable and rapidly degraded. We conclude that the rigid β-stem of the Hbp passenger should be left intact to ensure efficient extracellular expression through fast translocation kinetics and stability of the translocated fusion as a whole. These data are in agreement with a recent report showing that extracellular expression of heterologous proteins was improved when fused to the full-length passenger of the *Shigella* AT IcsA rather than to its cognate β-domain [23].

### Cell surface display of ESAT6

**Figure 3:**
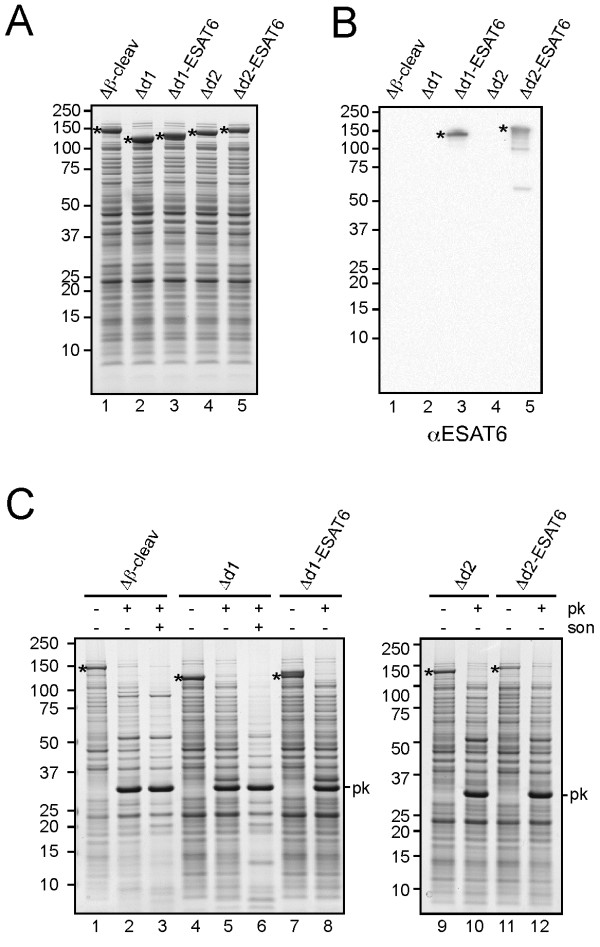
**Cell surface exposure of HbpD-ESAT6 fusions.** (**A-B**) Expression of Hbp display constructs analyzed by Coomassie staining (A) or immunoblotting (B) as described in the legend to Figure 1. (**C**) Proteinase K accessibility of HbpD-ESAT6 fusions at the cell surface. Part of the cells described under *A* was collected and resuspended in 50 mM Tris–HCl, pH 7.4, 1 mM CaCl. For Hbp(Δβ-cleav) and HbpD(Δd1), half of the cell suspension was lysed by sonication (*son*) on ice using a tip sonicator (Branson Sonifier 250). Subsequently, all samples were incubated with Proteinase K (*pk*; 100 μg/ml) at 37°C for 1 h. The reaction was stopped by addition of 0.1 mM phenylmethanesulfonylfluoride (PMSF) and incubation on ice for 5 min. Samples were TCA precipitated before analysis by SDS-PAGE and Coomassie staining. Non-cleaved Hbp species (*) are indicated. Molecular mass (kDa) markers are indicated at the left side of the panels.

Following OM translocation, the Hbp passenger is cleaved from its cognate β-domain through a conserved autocatalytic mechanism [24,25]. Previously we have created Hbp(Δβ-cleav) through genetic disruption of the inter-domain cleavage site. The passenger of this mutant is fully translocated to the cell surface where it remains covalently attached to its β-domain [17]. To test this Hbp construct as a platform for surface display of antigenic proteins, we created non-cleaved ‘display’ versions (HbpD) of the Hbp(Δd1)-ESAT6 and Hbp(Δd2)-ESAT6 chimeras (see Figure 1A; Additional 2: Figure S2). Initially, we confirmed proper expression by SDS-PAGE and Coomassie staining (Figure 3A). As a result of the absence of β-domain processing, these chimeras accumulated in the cell fraction with a ~30 kDa increase in apparent molecular mass compared to their cleaved counterparts (cf. Figure 3A and 1C). Immunoblotting was carried out to verify the presence of the Hbp passenger and β-domain (Additional file 4: Figure S4) as well as the ESAT6 antigen in the passenger domain (Figure 3B, lanes 3 and 5).

**Figure 4:**
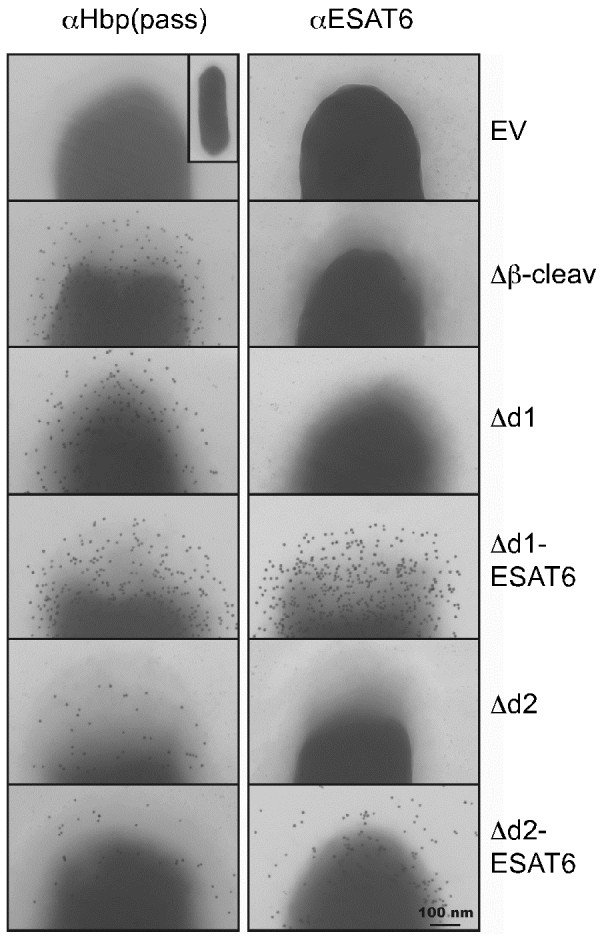
**Cell surface display of ESAT6 upon fusion to the Hbp passenger.** Cells expressing Hbp display constructs described in the legend to Figure 3A, and cells carrying an empty vector (EV), were fixed and analyzed by immuno-EM using Hbp passenger and ESAT6 specific antibodies as indicated. Scale bar: 100 nm.

As a first approach to confirm surface exposure, whole cells expressing the HbpD constructs, were treated with Proteinase K to digest extracellular proteins (Figure 3C). Clearly, all Hbp chimeras were specifically degraded. As a control for cell integrity, the periplasmic domain of the outer membrane protein (OMP) OmpA was not accessible unless the cells were lysed (Additional file 5: Figure S5). As a second approach to examine surface exposure, whole cells expressing the HbpD constructs were immobilized on a nitrocellulose membrane and detected by passenger and ESAT6 specific antibodies (Additional file 6: Figure S6A and S6B). As a control for cell integrity, the periplasmic protein OppA could only be detected upon cell lysis, (Additional file 6: Figure S6C). As a third approach, intact cells were subjected to immuno-electron microscopy (EM) analysis using either Hbp or ESAT6 antibodies (Figure 4). As opposed to cells carrying an empty vector (EV), all Hbp chimeras showed efficient and disperse labeling using anti-Hbp, confirming surface exposure of the respective passengers. Using anti-ESAT6, strong, dispersed surface labeling was observed for HbpD(Δd1)-ESAT6 and HbpD(Δd2)-ESAT6 expressing cells. In conclusion, efficient surface display of ESAT6 was achieved upon fusion to a non-cleavable Hbp variant.

### Secretion of ESAT6 upon fusion to the Autotransporter EspC

**Figure 5:**
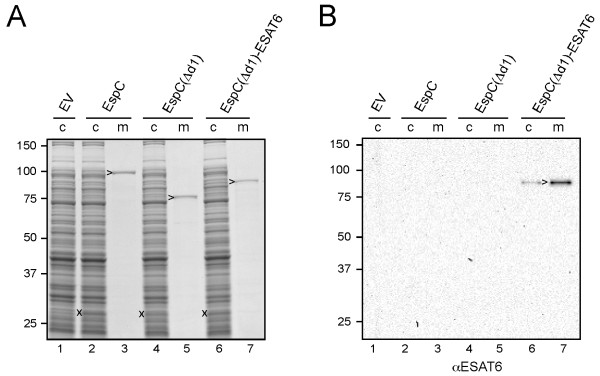
**Secretion of ESAT6 fused to the EspC passenger.** (**A-B**) Expression and secretion of EspC, EspC(Δd1), and EspC(Δd1)-ESAT6 analyzed by SDS-PAGE and Coomassie staining (A) or immunoblotting (B) as described in the legend to Figure 1. Cleaved passenger (>) is indicated. Molecular mass (kDa) markers are indicated at the left side of the panels.

The recent elucidation of several AT passenger structures [26-28] shows a highly conserved domain organization suggesting that our approach can be extended to other ATs. To test this supposition, we analyzed secretion of ESAT6 upon fusion to the passenger of the AT EspC from enteropathogenic *E. coli* using a similar approach as described above for Hbp. In a strategy that was guided by 3-dimensional homology modeling of the EspC passenger (Additional file 7: Figure S7A), EspC(Δd1), lacking side domain d1, and EspC(Δd1)-ESAT6, carrying ESAT6 at the position of d1, were created (Additional file 7: Figure S7B) and their secretion was analyzed SDS-PAGE and Coomassie staining (Figure 5A). Both constructs were efficiently expressed, processed and secreted into the culture medium (Figure 5A, lanes 4-7) at similar levels as wild-type EspC (Figure 5A, lanes 2-3). Furthermore, the presence of ESAT6 in secreted EspC(Δd1)-ESAT6 was shown by immunoblotting (Figure 5B, lane 7). These data demonstrate successful secretion of ESAT6 upon fusion to the EspC passenger. This suggests that our structurally informed strategy for extracellular expression of heterologous proteins is generally applicable to ATs.

### Extracellular transport of ESAT6 by attenuated *Salmonella typhimurium*

**Figure 6:**
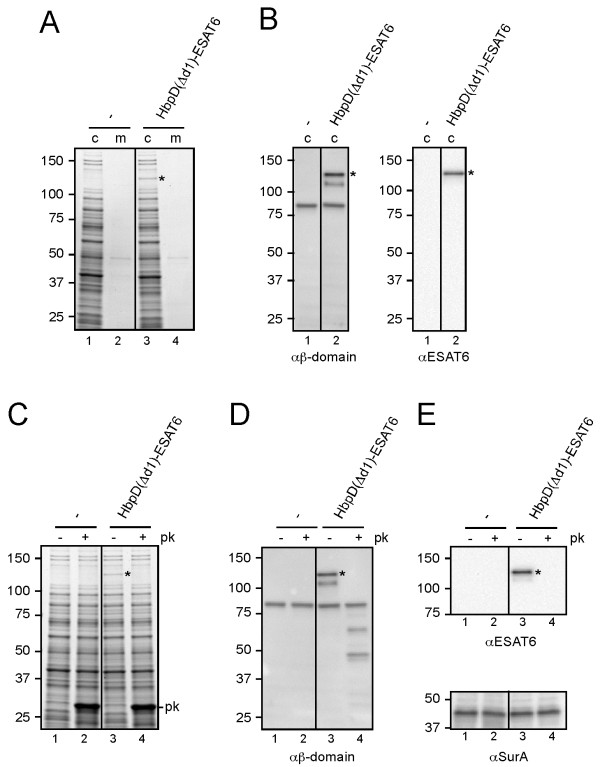
**Display of ESAT6 by attenuated *Salmonella typhimurium.*** (**A**-**B**) Expression of HbpD(Δd1)-ESAT6. *S. typhimurium* SL3261 (-) and a derivative expressing HbpD(Δd1)-ESAT6 were grown to mid-log phase in LB medium at 37°C. The equivalent of 0.03 OD_660_ units cells (c) and corresponding culture medium (m) samples was analyzed by SDS-PAGE and Coomassie staining (A) or immunoblotting (B). (**C**-**E**) Exposure of HbpD(Δd1)-ESAT6 at the *S. typhimurium* cell surface. (**C**) Cells from *A* were collected and resuspended in icecold reaction buffer (50 mM Tris HCl, pH 7.4, 1 mM CaCl_2_). The samples were treated with 100 μg/ml Proteinase K (*+ pk*) or mock-treated (*- pk*) at 37°C for 1 h. The reaction was stopped by incubation with PMSF (0.1 mM) for 10 min on ice. Samples were TCA precipitated and analyzed as described under *A*. (**D**-**E**) Samples described under *C* were analyzed by immunoblotting. Cell integrity during the procedure was demonstrated by showing the inaccessibility of the periplasmic chaperone SurA towards Proteinase K using anti-SurA (cf. lanes 1, 3, 5 and 2, 4, 6, resp.). Non-cleaved Hbp species (*) are indicated. Molecular mass (kDa) markers are shown at the left side of the panels.

Live attenuated strains of pathogenic bacteria that synthesize foreign antigens are being developed as vaccines for several infectious diseases and cancer. Attenuated derivatives of *S. typhimurium* have been most extensively studied for this purpose because it is a facultative intracellular bacterium that provokes a strong cellular immune response [29]. Using *Salmonella* vaccine strains, extracellular expression of antigens has been shown to improve CD4^+^ and CD8^+^ T cell responses compared to intracellular expression [30,31]. Since AT systems, in general, function in heterologous Gram-negative hosts [32-34] we have explored the potential of Hbp as a platform for live vaccine development by examining the display of the HbpD(Δd1)-ESAT6 fusion in the attenuated *S. typhimurium* strain SL3261 [35] (Figure 6). For stable expression, a single copy of the gene encoding the chimera was integrated into the genome and placed under control of a *lac*UV5 promoter, which is constitutively expressed in *Salmonella*.

Despite the single copy background, substantial amounts of HbpD(Δd1)-ESAT6 were detected in the cell fraction of *Salmonella* using SDS-PAGE and Coomassie staining (Figure 6A, lane 3). The presence of both the β-domain and the ESAT6 antigen in the display construct was confirmed by immunoblotting (Figure 6B). As expected, HbpD(Δd1)-ESAT6 was exposed at the cell surface judged by its sensitivity to externally added Proteinase K (Figure 6C, D, E, lane 4). Cell integrity during the procedure was confirmed by the inaccessibility of the periplasmic chaperone SurA towards Proteinase K (Figure 5E, lane 4). Together, the data underscore the potential of Hbp as a platform for the development of recombinant bacterial live vaccines.

For surface display, antigen fragments have been inserted in surface exposed proteins like OMPs and fimbriae [36] whereas secretion has been achieved via the type I-III secretion pathways [1]. However, these systems are limited in the size and complexity of the antigens that can be accommodated and in the yields of extracellular antigen. Here, we have modified the Hbp into a vaccine carrier that can display the full-length mycobacterial antigen ESAT6 with great efficiency in a live attenuated *Salmonella* strain. Fusion to the intact ~100 Å long β-stem [9] has the additional advantage of optimal presentation of antigens to the immune system at some distance from the cell surface. Notably, the Hbp passenger possesses multiple sites for the insertion of heterologous sequences (see Figure 1), suggesting that various antigens could be fused to same Hbp β-stem. This would enable the formation of a multivalent recombinant live vaccine, which would be highly valuable since multivalency appears an important feature in the protection against infectious diseases such as TB [10]. Importantly, since the passenger side domains carry the functionality of Hbp [18,37] their replacement by antigenic proteins automatically eliminates potential toxic effects, making the presented Hbp platform safe to use for vaccination.

## Conclusions

Whereas previous attempts to exploit ATs suffered from a lack of structural information, we took advantage of the available crystal structure of the Hbp passenger to build a platform for the secretion or display of recombinant proteins. We show that replacement of Hbp passenger side domains is a successful strategy to achieve high-level secretion and display of ESAT6 in *E. coli*, and that the presence of an intact Hbp β-stem is important for optimal secretion and stability of Hbp-ESAT6 chimeras. ESAT6 was also successfully secreted substituting a passenger side domain of the AT EspC, demonstrating a more general applicability of the approach. Furthermore, efficient display of Hbp-ESAT6 was shown in an attenuated *Salmonella typhimurium* strain, demonstrating the potential for the development of live bacterial vaccines. Of note, the presented platform could also be of value for other industrial applications that require high-level secretion or display of heterologous proteins.

## Methods

### *E. coli* strains and culturing conditions

*E. coli* strain MC1061 was used for protein production. Cells were grown at 37°C in LB medium containing 0.2% glucose and the antibiotics chloramphenicol (30 μg/ml) and streptomycin (25 μg/ml).

### Reagents and sera

Restriction enzymes, alkaline phosphatase & DNA ligase (Rapid DNA Dephos & Ligation Kit), Lumi-light Western blotting substrate and Proteinase K (recombinant, PCR grade) were from Roche Applied Science. Phusion DNA polymerase was from Finnzymes. EM-grade paraformaldehyde and glutaraldehyde were from Electron Microscopy Sciences. The polyclonal antisera against the Hbp passenger (J40) and β-domain (SN477) have been described previously [19,38]. Monoclonal antibodies against ESAT6 (HYB 76-8) have been described previously [16]. The polyclonal antiserum against OmpA was from our own labcollection. The polyclonal antiserum against OppA was a gift from K. Igarashi (Chiba University, Japan), whereas the antiserum against SurA was a gift from T. Silhavy (Princeton University, USA).

### Plasmid construction

All expression plasmids used have a pEH3 [39] backbone. The pHbp plasmids below were all based on pEH3-Hbp(ΔBamHI), a pEH3-Hbp [17] derivative lacking the BamHI site downstream of the *hbp* ORF. For display purposes, pHbpD plasmids were generated based on pEH3-HbpD(ΔBamHI), a pEH3-Hbp(Δβcleav) [17] derivative lacking the BamHI site downstream of the *hbp* ORF. A second BamHI site, at the junction of the passenger and β-domain coding sequences of pEH3-Hbp(Δβcleav), was removed by overlap-extension PCR using the mutagenesis primers Hbp BamHI QC fw and Hbp BamHI QC rv.

Plasmids pHbp(Δd1), pHbp(Δd2), pHbp(Δd3), pHbp(Δd4) and pHbp(Δd5) were created upon substitution of passenger subdomain coding sequences of pEH3-Hbp(ΔBamHI) by a Gly/Ser encoding linker sequence containing SacI and BamHI restriction sites using overlap-extension PCR. To substitute domain 1, the primers used were Hbp(Δd1) fw and Hbp(Δd1) rv, yielding pHbp(Δd1). To substitute domain 2, the primers used were Hbp(Δd2) fw and Hbp(Δd2) rv, yielding pHbp(Δd2). To substitute domain 3, the primers used were Hbp(Δd3) fw and Hbp(Δd3) rv, yielding pHbp(Δd3). To substitute domain 4, the primers used were Hbp(Δd4) fw and Hbp(Δd4) rv, yielding pHbp(Δd4). To substitute domain 5, the primers used were Hbp(Δd5) fw and Hbp(Δd5) rv, yielding pHbp(Δd5). Using the same strategy, plasmids pHbpD(Δd1) and pHbpD(Δd2) were created, but based on pEH3-HbpD(ΔBamHI). To create pHbpD(Δd1), the primers used were Hbp(Δd1) fw and Hbp(Δd1) rv. To construct pHbpD(Δd2), the primers used were Hbp(Δd2) fw and Hbp(Δd2) rv.

To insert the coding sequence for ESAT6 into the pHbp and pHbpD derivatives described above, an *E. coli*-codon-usage-optimized synthetic gene of *M. tuberculosis* gene *esxA* was constructed by Baseclear B.V. The synthetic gene was flanked by 5’-gagctcc-3’ and 5’-ggatcc-3’ sequences at the 5’ and 3’ site, respectively, allowing in-frame insertion into the *hbp* ORF of the pHbp and pHbpD derivatives using the SacI/BamHI restriction sites. This approach was used to construct pHbp(Δd1)-ESAT6, pHbp(Δd2)-ESAT6, pHbp(Δd3)-ESAT6, pHbp(Δd4)-ESAT6, pHbp(Δd5)-ESAT6, pHbpD(Δd1)-ESAT6 and pHbpD(Δd2)-ESAT6.

To construct plasmids pHbp(Tr557)-ESAT6, pHbp(Tr804)-ESAT6, pHbp(Tr868)-ESAT6 and pHbp(Tr942)-ESAT6, plasmids pHbp(Tr557), pHbp(Tr804), pHbp(Tr868) and pHbp(Tr942) were constructed first. To construct pHbp(Tr557) a PCR fragment was created using pEH3-Hbp(ΔBamHI) as a template and the primers Hbp(Tr557) fw and Hbp EcoRI rv. The resulting fragment was cloned into pHbp(Δd1) using the BamHI/EcoRI restriction sites yielding pHbp(Tr557). pHbp(Tr804), pHbp(Tr868) and pHbp(Tr942) were created using the same strategy except that Hbp(Tr804) fw Hbp(Tr868) fw and Hbp(Tr942) fw were used as the forward primer, respectively. Subsequently, pHbp(Tr557)-ESAT6, pHbp(Tr804)-ESAT6, pHbp(Tr868)-ESAT6 and pHbp(Tr942)-ESAT6 were created by inserting the synthetic *esxA* gene decribed above using the SacI/BamHI restriction sites.

To create pEH3-EspC(Δd1), a three-step overlap-extension PCR procedure was carried out. In the first step a DNA fragment was amplified by PCR using pEH3-EspC [40] as a template and the primers pEH_XbaI_EspC fw and EspC(Δdom1/Cas) rv. In the second step a DNA fragment was amplified by PCR using pEH3-EspC as a template and the primers EspC(Δdom1/Cas) fw and EspC(BglII) rv. In the third step a DNA fragment was amplified using a mixture of the PCR products from step 1 and 2 as template and the primers pEH_XbaI_EspC fw and EspC(BglII) rv. The PCR product from step 3 was cloned into pEH3-EspC using the XbaI and BglII restriction sites, yielding plasmid pEH3-EspC(Δd1). Plasmid pEH3-EspC(Δd1)-ESAT6 was created by inserting the synthetic *esxA* gene decribed above using the SacI/BamHI restriction sites.

To construct pHbp(Δd1)-hEGF a synthetic DNA sequence encoding hEGF (accession number Q6QBS2) was constructed by Baseclear B.V. The synthetic gene was flanked by 5’-gagctcc-3’ and 5’-ggatcc-3’ sequences at the 5’ and 3’ site, respectively, allowing cloning into the SacI and BamHI sites of pHbp(Δd1), yielding pHbp(Δd1)-hEGF. The same strategy was used to construct pHbp(Δd1)-hEGF(0ss) except that a DNA fragment was used in which the cysteine codons had been replaced by serine codons.

Nucleotide sequences of all constructs were confirmed by semi-automated DNA sequencing. Primer sequences are listed in Table 1.

**Table 1:** Primers used in this study

**Primer**	**Sequence (5’ → 3’)**
Hbp(down XmaI) rv	gtaccgacaaattctccctg
Hbp BamHI QC fw	cttcatcactgaagttggttccctgaacaaacgcatg
Hbp BamHI QC rv	catgcgtttgttcagggaaccaacttcagtgatgaag
Hbp(Δd1) fw	gggagctcctgcggatccggcagcggtaatgatg ccccggtcacgttc
Hbp(Δd1) rv	cggatccgcaggagctccccgcaagacttcctgc agag
Hbp(Δd2) fw	ctgggagctccgcaggatccggcagcggtaatac tgcagggtatctgtttc
Hbp(Δd2) rv	ctgccggatcctgcggagctcccagaaccggcata gtccagcgtgatag
Hbp(Δd3) fw	gggagcgggagctccgcaggatccggcagcgg taaccgcagttttacctttgac
Hbp(Δd3) rv	accgctgccggatcctgcggagctcccgctcccct gcagcgtcagacg
Hbp(Δd4) fw	gggagcgggagctccgcaggatccggcagcggt agtgtcttcaacggcaccg
Hbp(Δd4) rv	accgctgccggatcctgcggagctcccgctcccgtc gcccagcgtgacgctg
Hbp(Δd5) fw	gggagcgggagctccgcaggatccggcagcggg taccgcaatatctggagc
Hbp(Δd5) rv	gctgccggatcctgcggagctcccgctccctccgag ggtgacagtc
Hbp(Tr557) fw	gcggggagctccgcaggatccggcagcggtgcag ggtatctgtttcacgg
Hbp(Tr804) fw	gcggggagctccgcaggatccggcagcggtttacc tgtatacgattatgccg
Hbp(Tr868) fw	gcggggagctccgcaggatccggcagcgggtacc gcaatatctggagcgg
Hbp(Tr942) fw	gggagctcctgcggatccggcagcggtgcagacaa actggtgataaac
Hbp EcoRI rv	cagtgaattctcagaatgaataacgaatattag
lacUV5_ScaI_fw	gcgcagtactttgcgccattctatggtgtc
pEH3Hbpβ_ScaI_rv	gcgcagtactcacagcatcagaatgaataacg
malE_insert_seq	tataacccttgtcgccgttg
malK_insert_seq	acgcagcaaggtcgatttac
pEH_XbaI_EspC_fw	taactttctagattacaaaacttaggagggtttttacca tgaataaaatatacgcattaaaata
EcoRI_EspC rv	gtcagaattctcagaaagaataacggaagttag
EspC(Δdom1/Cas) fw	gggagctccgcaggatccggcagcggtttaaaaaa caaatttactcaaaaagtc
EspC(Δdom1/Cas) rv	cggatcctgcggagctcccagcctgagatgcgctta aaaaag
EspC (BglII) rv	ccagagccaatgtttacgtc

### Construction of *S. typhimurium* strains

The *hbp* gene and its mutant derivatives were inserted into the chromosome of *S. typhimurium* by allelic exchange through double cross-over homologous recombination [41], replacing the *malE* and *malK* promotor regions. Briefly, *hbp* including the *lac*UV5 promoter region was amplified by PCR from pEH3-Hbp using primers lacUV5_ScaI_fw and pEH3Hbpbeta_ScaI_rv. The PCR product was digested with ScaI and cloned into a SmaI cut pSB890-derived suicide vector [42], just in between 1000 bp homology regions to *malE* and *malK*. The *hbp*-suicide vector was transformed into the *E. coli* donor strain SM10λ *pir*[43]. SM10λ *pir* was mated overnight on plate with the *Salmonella* recipient strain SL3261 [35]. Tetracyclin resistant *Salmonella* transconjugants were selected on plate. Resolution of merodiploids and replacement of the wild-type locus with *hbp* were achieved by selecting for resistance of the *Salmonella* mutants to sucrose [41]. Positive clones were identified by PCR of the intergenic region between *malE* and *malK* using primers malE_insert_seq and malK_insert_seq and sequencing of the introduced allele. Primer sequences are listed in Table 1.

### Whole cell labeling by immuno-EM

Cultures were grown and induced for 2 h as described above. Cells were collected and resuspended in 0.9% NaCl, after which they were fixed by addition of an equal volume of Fixation solution (0.4 M PHEM buffer, 4% paraformaldehyde, 0.2% glutaraldehyde) and incubation for 2 h at room temperature. The fixed cells were harvested by low-speed centrifugation (1,500 x *g* for 5 min), resuspended in Storage solution (0.1 M PHEM buffer, 0.5% paraformaldehyde) and kept at 4°C until further analysis. For immuno-labeling of whole mount cells, formvar-carbon coated copper grids were floated on small drops of fixed cells for 5 min at room temperature. Subsequently, the grids were washed three times on drops of PBS and blocked with PBS-BSA 1% for 3 min. Next, the grids were incubated for 1 h with antibodies that were diluted in PBS supplemented with 1% BSA. After 5 washes with PBS, the antibodies were labeled with rabbit anti-mouse bridging serum (DAKO; 1:100) (if monoclonal), probed with protein-A conjugated to 10 nm gold (EM laboratory, Utrecht University) and imaged on a CM 10 microscope.

### General protein expression and analysis

In *E. coli*, the ORFs encoding Hbp(derivatives) or EspC(derivatives) were expressed from vector pEH3 under control of a *lac*UV5 promoter [39]. When cultures reached an OD_660_ of ~ 0.3, cells were induced for protein production by the addition of IPTG (1 mM) and growth was continued for 2 h. For both *E. coli* and *S. typhimurium*, culture samples were withdrawn and separated in cells and spent medium by low speed centrifugation, and analyzed by SDS-PAGE followed by Coomassie (G-250) staining or immunoblotting. Cells were resuspended in SDS-sample buffer (125 mM Tris–HCl, pH 6.8, 4% SDS, 20% glycerol, 0.02% bromophenol blue, 100 mM DTT) directly whereas medium samples were first trichloroacetic acid (TCA)-precipitated. Quantification of immunoblot signals was carried out using Quantity One software (Biorad).

## Abbreviations

AT: Autotransporter; IM: inner membrane; OM: outer membrane; IPTG: isopropyl β-D-1-thiogalactopyranoside; AC: autochaperone; EM: electron microscopy; OMP: outer membrane protein; PMSF: phenylmethanesulfonylfluoride; TCA: trichloroacetic acid.

## Competing interests

JWdG and SW are co-founders of Xbrane Bioscience AB that aims to exploit the presented Hbp platforms for commercial protein production. JL is consultant for and WSPJ and DW are (in part) employed by Xbrane Bioscience AB.

## Authors' contributions

W.S.P.J., Z.S., K.d.P., C.t.H.J., S.W. and D.W. performed research; W.S.P.J., Z.S., S.W., D.W., J.W.d.G., N.v.d.W. and J.L. analyzed data. P.A. contributed essential reagents. W.S.P.J. and J.L. designed research and wrote the manuscript. All authors read and approved the final manuscript.

## Supplementary Material

Additional file 1 **Supplemental Figure S1. **Side domains of the Hbp passenger domain. Click here for file

Additional file 2 **Supplemental Figure S2. **Schematic representation of Hbp derivatives used in the study. Click here for file

Additional file 3 **Supplemental Figure S3. **Secretion of hEGF and cysteineless hEGF(0ss) fused to the Hbp passenger. Click here for file

Additional file 4 **Supplemental Figure S4.** Immunoblots of HbpD-ESAT6 expression. Click here for file

Additional file 5 **Supplemental Figure S5. **Sensitivity of OmpA towards Proteinase K in cells expressing HbpD-ESAT6 fusions. Click here for file

Additional file 6 **Supplemental Figure S6. **Display of ESAT6 at the cell surface. Click here for file

Additional file 7 **Supplemental Figure S7. **Cartoons EspC-ESAT6 fusion. Click here for file
