# Supplementary material for: A structurally informed autotransporter platform for efficient heterologous protein secretion and display
Source: Microb Cell Fact. 2012 Jun 18;11:85. doi: 10.1186/1475-2859-11-85 (PMC3521207; doi:10.1186/1475-2859-11-85)
Supplement: Additional file 1 — Supplemental Figure S1. Side domains of the Hbp passenger domain. [file 1475-2859-11-85-S1.pdf]

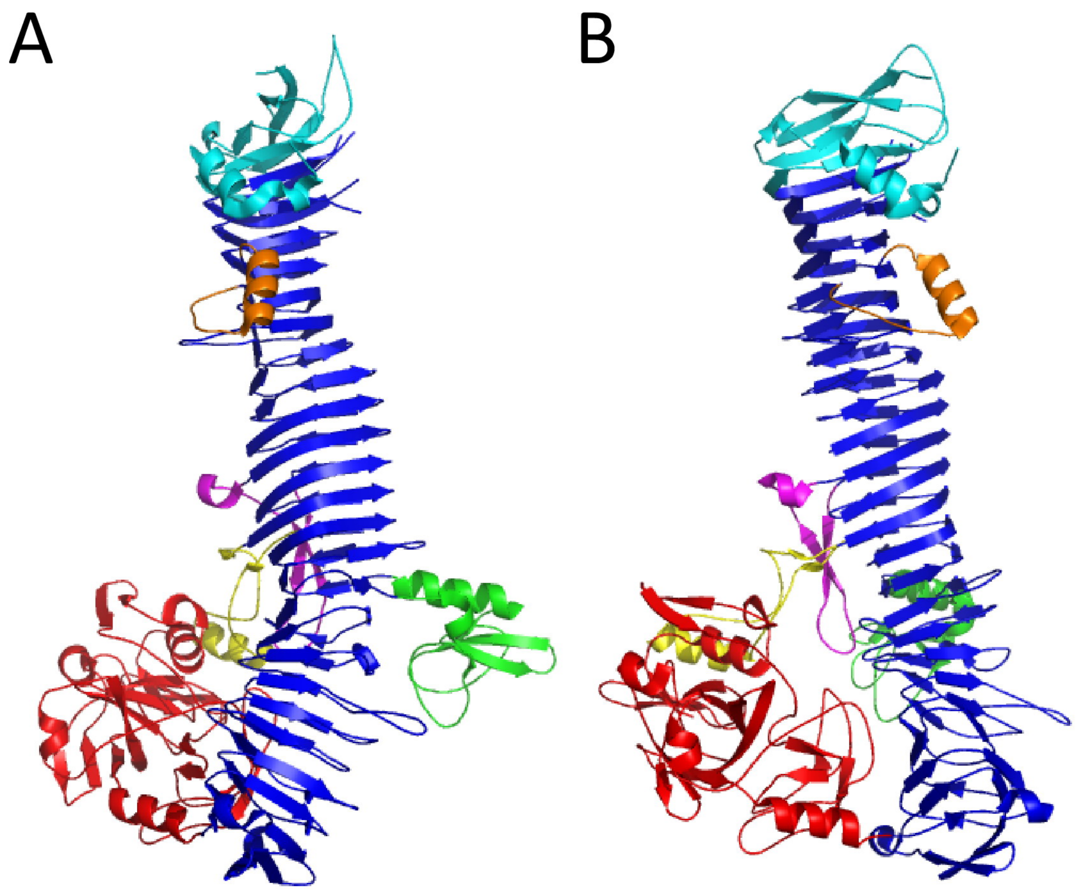

Fig. S1. Side domains of the Hbp passenger domain. (A) Cartoon of the crystal structure of the passenger domain of the *E. coli* AT Hbp [PDB: 1WXR]. The side domains d1 (*red*), d2 (*green*), d3 (*yellow*), d4 (*magenta*), d5 (*orange*), and the conserved autochaperone domain (*cyan*) are highlighted. The remainder of the passenger, including the β-stem domain, is in dark blue. **(B)** Cartoon of the crystal structure as in *A* rotated around the y-axis (50°counter clockwise). The image was created using MacPyMol.
