# Supplementary material for: A structurally informed autotransporter platform for efficient heterologous protein secretion and display
Source: Microb Cell Fact. 2012 Jun 18;11:85. doi: 10.1186/1475-2859-11-85 (PMC3521207; doi:10.1186/1475-2859-11-85)
Supplement: Additional file 2 — Supplemental Figure S2. Schematic representation of Hbp derivatives used in the study. [file 1475-2859-11-85-S2.pdf]

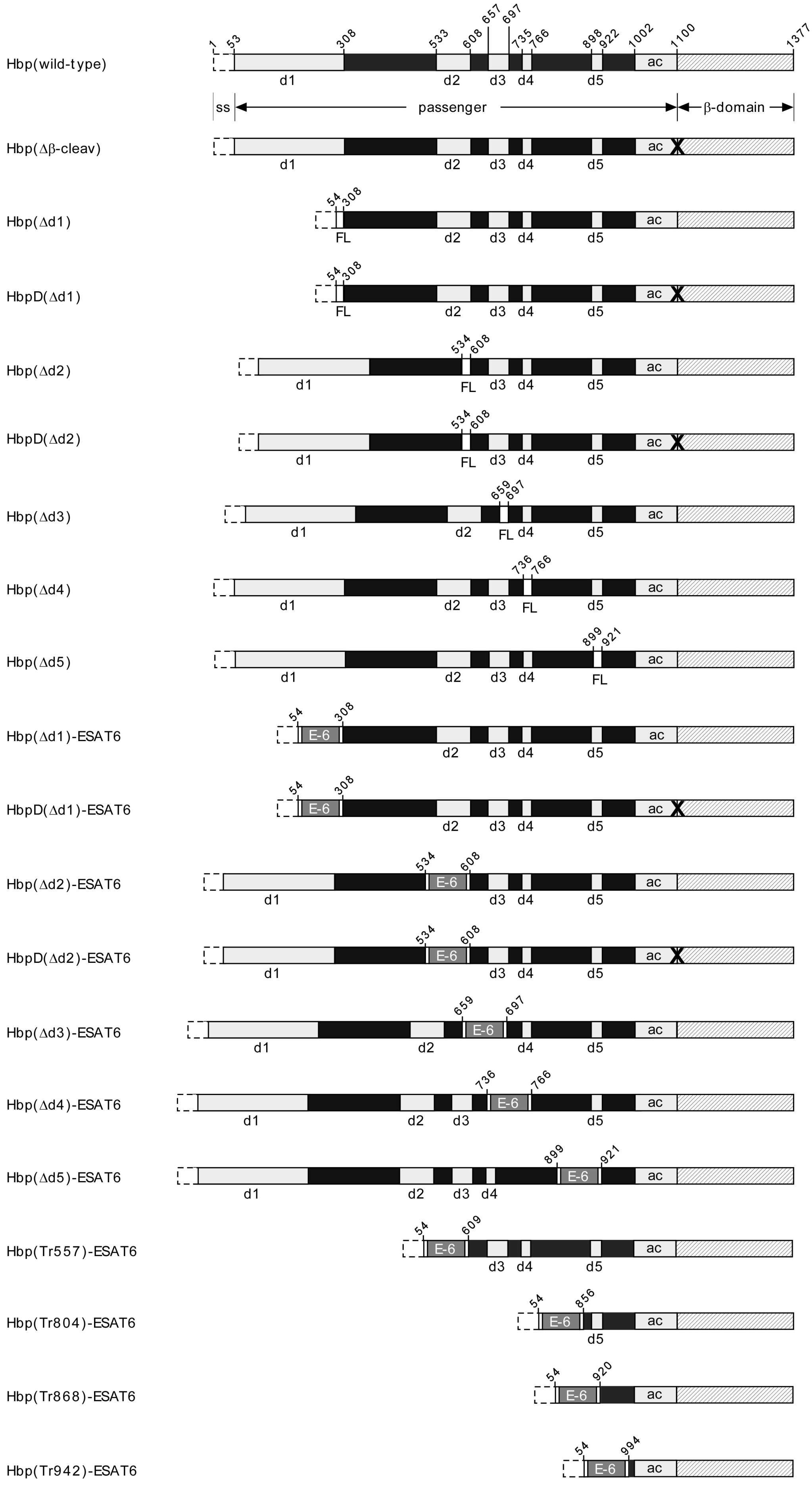

Hbp(wild-type)
1
53
308
533
608
657
697
735
766
898
922
1002
1100
1377
d1
d2
d3
d4
d5
ac
ss
passenger
β-domain
Hbp(Δβ-cleav)
Hbp(Δd1)
54
308
FL
HbpD(Δd1)
Hbp(Δd2)
534
608
HbpD(Δd2)
Hbp(Δd3)
659
697
Hbp(Δd4)
736
766
Hbp(Δd5)
899
921
Hbp(Δd1)-ESAT6
E-6
HbpD(Δd1)-ESAT6
Hbp(Δd2)-ESAT6
HbpD(Δd2)-ESAT6
Hbp(Δd3)-ESAT6
Hbp(Δd4)-ESAT6
Hbp(Δd5)-ESAT6
Hbp(Tr557)-ESAT6
609
Hbp(Tr804)-ESAT6
856
Hbp(Tr868)-ESAT6
920
Hbp(Tr942)-ESAT6
994

Fig. S2. Schematic representation of Hbp derivatives used in the study. Hbp is synthesized as a 1377 amino acid (*aa*) precursor that is organized in three domains: (i) an N-terminal cleavable signal sequence (*ss*; aa 1-52), (ii) a secreted passenger domain (aa 53-1100) and (iii) an OM integrated C-terminal β-domain (aa 1101-1377). Side domains d1-d5 and the autochaperone domain (*ac*) of the passenger domain are indicated. The remainder of the passenger domain, including the β-stem is in black. After passage of the OM, the passenger is cleaved from the β-domain via an autocatalytic mechanism that involves hydrolysis of the peptide bond between two asparagines at position 1100 and 1101 of the Hbp precursor [1, 2]. Substitution of these asparagines by a glycine and serine, respectively, prevents cleavage [3], denoted ***X***. Numbers displayed above the diagrams correspond to the amino acid positions of the wild-type Hbp precursor, calculated from the N-terminus. Insertion of a 9-11 amino acid long flexible linker (*FL*) comprising glycine and serine residues, as well as insertion of the mycobacterial antigen ESAT6 (*E-6*), is indicated.

References

1. Dautin N, Barnard TJ, Anderson DE, Bernstein HD: Cleavage of a bacterial autotransporter by an evolutionarily convergent autocatalytic mechanism. *EMBO J* 2007, 26(7):1942-1952.
2. Roussel-Jazede V, Van Gelder P, Sijbrandi R, Rutten L, Otto BR, Luirink J, Gros P, Tommassen J, Van Ulsen P: Channel properties of the translocator domain of the autotransporter Hbp of Escherichia coli. *Mol Membr Biol* 2011, 28(3):158-170.
3. Jong WS, ten Hagen-Jongman CM, den Blaauwen T, Slotboom DJ, Tame JR, Wickstrom D, de Gier JW, Otto BR, Luirink J: Limited tolerance towards folded elements during secretion of the autotransporter Hbp. *Molecular microbiology* 2007, 63(5):1524-1536.
