# Supplementary material for: A structurally informed autotransporter platform for efficient heterologous protein secretion and display
Source: Microb Cell Fact. 2012 Jun 18;11:85. doi: 10.1186/1475-2859-11-85 (PMC3521207; doi:10.1186/1475-2859-11-85)
Supplement: Additional file 3 — Supplemental Figure S3. Secretion of hEGF and cysteineless hEGF(0ss) fused to the Hbp passenger. [file 1475-2859-11-85-S3.pdf]

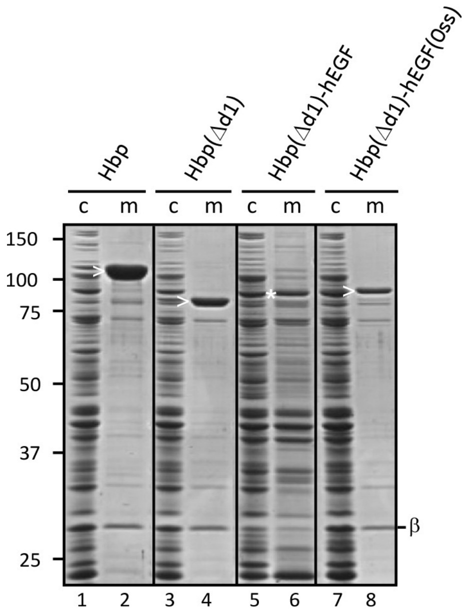

Fig. S3. Secretion of hEGF and cysteineless hEGF(0ss) fused to the Hbp passenger. Secretion of Hbp, Hbp(Δd1), Hbp(Δd1)-hEGF and Hbp(Δd1)-hEGF(0ss) analyzed by SDS-PAGE and Coomassie staining as described in the legend to Fig. 1, except that cells were grown in M9 medium supplemented with 0.2% glucose. The equivalent of 0.05 OD_{660} units of cells (c) and 0.1 OD_{660} units of spent medium (m) was analyzed. A band unrelated to Hbp-hEGF that was released into the culture medium due to cell lysis is indicated (*). The identity of fused hEGF and hEGF(0ss) was confirmed by immunoblotting (data not shown). Secreted passengers (>) and the cleaved β-domain (β) are indicated. Molecular mass (kDa) markers are indicated at the left side of the panels.
