# Supplementary material for: A structurally informed autotransporter platform for efficient heterologous protein secretion and display
Source: Microb Cell Fact. 2012 Jun 18;11:85. doi: 10.1186/1475-2859-11-85 (PMC3521207; doi:10.1186/1475-2859-11-85)
Supplement: Additional file 4 — Supplemental Figure S4. Immunoblots of HbpD-ESAT6 expression. [file 1475-2859-11-85-S4.pdf]

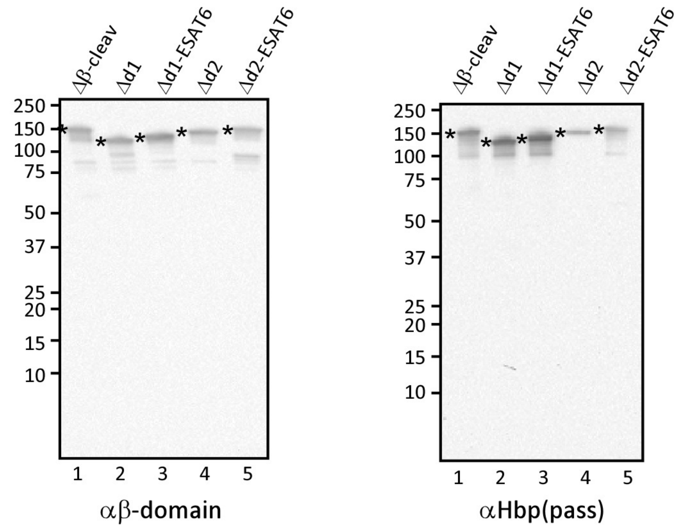

Fig. S4. Immunoblots of HbpD-ESAT6 expression. Cells described in the legend to Fig. 3A were analyzed by immunoblotting using Hbp passenger and β-domain specific antisera. Non-cleaved Hbp species (*) are indicated. Molecular mass (kDa) markers are indicated at the left side of the panels.
