# Supplementary material for: A structurally informed autotransporter platform for efficient heterologous protein secretion and display
Source: Microb Cell Fact. 2012 Jun 18;11:85. doi: 10.1186/1475-2859-11-85 (PMC3521207; doi:10.1186/1475-2859-11-85)
Supplement: Additional file 5 — Supplemental Figure S5. Sensitivity of OmpA towards Proteinase K in cells expressing HbpD-ESAT6 fusions. [file 1475-2859-11-85-S5.pdf]

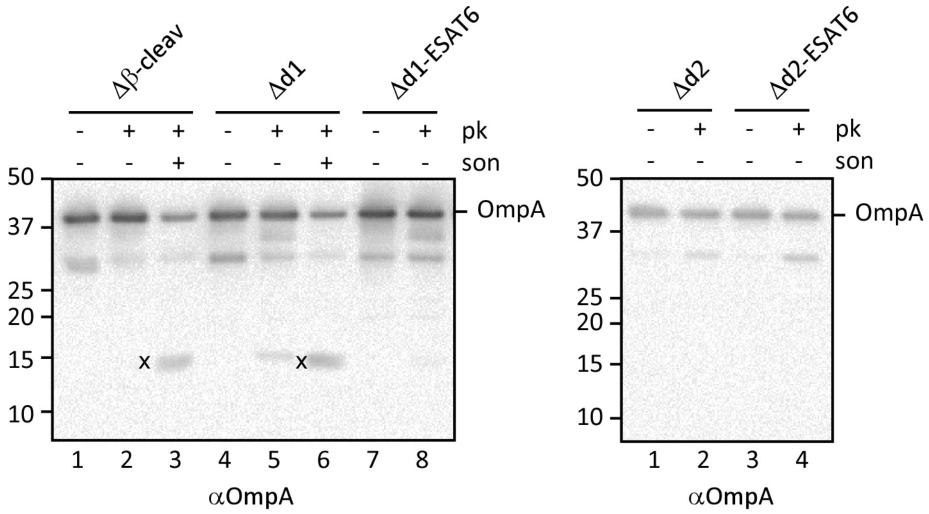

Fig. S5. Sensitivity of OmpA towards Proteinase K in cells expressing HbpD-ESAT6 fusions. Cells described in the legend to Fig. 3C were analyzed by immunoblotting using polyclonal antibodies directed against the OM protein OmpA, which is naturally inaccessible to Proteinase K unless cells are lysed. An OmpA degradation product that emerges upon Proteinase K treatment is indicated (*x*). Molecular mass (kDa) markers are indicated at the left side of the panels.
