# Supplementary material for: A structurally informed autotransporter platform for efficient heterologous protein secretion and display
Source: Microb Cell Fact. 2012 Jun 18;11:85. doi: 10.1186/1475-2859-11-85 (PMC3521207; doi:10.1186/1475-2859-11-85)
Supplement: Additional file 6 — Supplemental Figure S6. Display of ESAT6 at the cell surface. [file 1475-2859-11-85-S6.pdf]

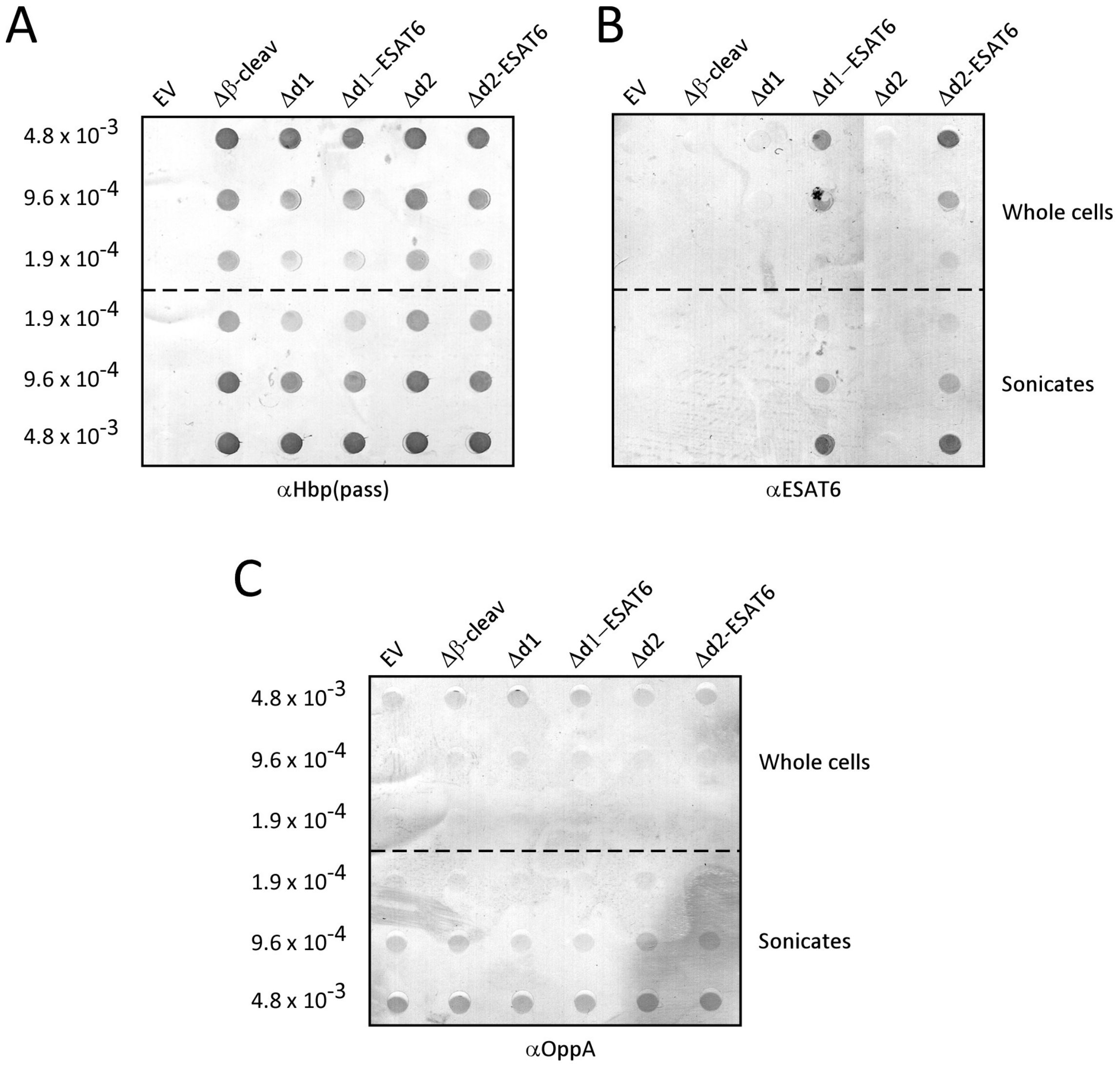

Fig. S6. Display of ESAT6 at the cell surface. (A) *E. coli* MC1061 cells carrying an empty vector (*EV*), or a plasmid encoding Hbp(Δβ-cleav), HbpD(Δd1), HbpD(Δd1)-ESAT6, HbpD(Δd2) or HbpD(Δd2)-ESAT6 were grown to early log phase in LB at 37°C and expression of Hbp derivatives was induced with 1 mM of IPTG. Cells were collected 1h after induction by low-speed centrifugation, washed and resuspended in ice-cold 50 mM Tris-HCl, pH 7.4 and stored on ice. Half of each sample was subjected to tip sonication on ice (Branson Sonifier 250) to lyse the cells, whereas the other half remained untreated. Subsequently, a five-fold dilution range of each sample was prepared in ice-cold 50 mM Tris-HCl, pH 7.4. Dilutions of each sample were applied to presoaked nitrocellulose membranes using a vacuum manifold Bio-Dot apparatus (Biorad). Membranes were blocked by incubation in 5% skimmed milk in Tris-Buffered Saline (TBS) for 20 min. To detect surface exposure of the passenger of Hbp derivatives, membranes were incubated with antiserum against the Hbp passenger in TBS. The membranes were washed with TBS before incubation with HRP-conjugated goat anti-rabbit IgG in TBS. The membranes were washed again with TBS and developed using di-octylsodiumsulphosuccinate (DONS) staining. **(B)** To detect surface exposure of ESAT6, cells were prepared and treated as under *A* except that mouse monoclonal antibodies directed against ESAT6 were used and HRP-conjugated rabbit anti-mouse IgG. **(C)** Cells were prepared and treated as under *A* except that a rabbit polyclonal antiserum against the periplasmic protein OppA was used as a control for cell integrity. At the left hand side of the panels the amount of material (in OD_{660} units) applied is indicated.
