# Supplementary material for: A structurally informed autotransporter platform for efficient heterologous protein secretion and display
Source: Microb Cell Fact. 2012 Jun 18;11:85. doi: 10.1186/1475-2859-11-85 (PMC3521207; doi:10.1186/1475-2859-11-85)
Supplement: Additional file 7 — Supplemental Figure S7. Cartoons EspC-ESAT6 fusion. [file 1475-2859-11-85-S7.pdf]

A B

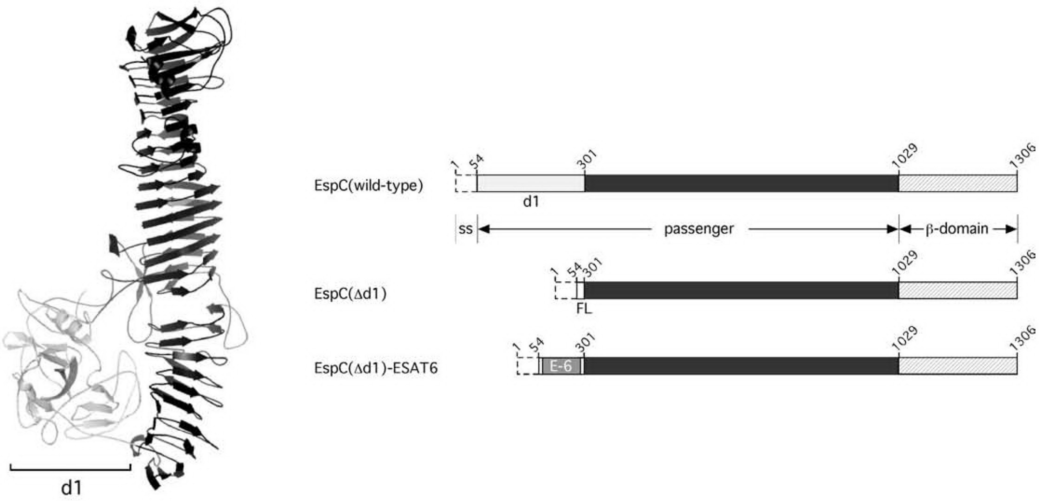

Fig. S7. Cartoons EspC-ESAT6 fusion. (A) Cartoon of a structural model of the passenger domain of EspC [Swiss-Prot: Q9EZE7] generated using M4T homology modeling [1]. Side domain 1 (*d1*) is in light grey. The remainder of the passenger domain, including the β-stem domain, is in black. **(B)** Schematic representation of EspC derivatives used. Wild-type EspC is synthesized as a 1306 amino acid (aa) precursor that is organized in three domains: (i) an N-terminal cleavable signal sequence (*ss*) (aa 1-53), (ii) a passenger domain (aa 54-1028) and (iii) an OM integrated β-domain (aa 1029-1306). After passage of the OM, the passenger is cleaved from the β-domain via an autocatalytic mechanism that involves hydrolysis of the peptide bond between two asparagines at position 1028 and 1029 of the EspC precursor [2]. The predicted domain d1 is indicated. The remainder of the passenger domain, including the β-stem domain, is in black. "FL" denotes flexible linker. "E-6" indicates ESAT6.

References

1. Rykunov D, Steinberger E, Madrid-Aliste CJ, Fiser A: Improved scoring function for comparative modeling using the M4T method. *J Struct Funct Genomics* 2009, 10(1):95-99.
2. Dautin N, Barnard TJ, Anderson DE, Bernstein HD: Cleavage of a bacterial autotransporter by an evolutionarily convergent autocatalytic mechanism. *EMBO J* 2007, 26(7):1942-1952.
